# Contribution of Mesenchymal Stem Cells from Obese Adipose Tissue to PD-L1 Over-Expression and Breast Cancer Progression through Pathogenic Th17 Cell Activation

**DOI:** 10.3390/cancers15112963

**Published:** 2023-05-29

**Authors:** Ferdinand Blangero, Maud Robert, Thomas Andraud, Charles Dumontet, Hubert Vidal, Assia Eljaafari

**Affiliations:** 1 CarMeN Laboratory, INSERM U1060, INRAE U1397, University Claude Bernard Lyon 1, Hospices Civils de Lyon, Centre Hospitalier Lyon Sud, 69310 Pïerre Bénite, France; 2 Bariatric Surgery Department, Edouard Herriot Hospital, 69003 Lyon, France; 3 Center of Research in Cancerology of Lyon, INSERM U1052, CNRS 5286, University Claude Bernard Lyon 1, Centre Léon Berard, 69008 Lyon, France; 4 Research Department, Hospices Civils de Lyon, 69002 Lyon, France

**Keywords:** adipose-tissue-derived mesenchymal stem cells, obesity, breast cancer, pathogenic Th17 cells, IFNγ, IL-17, immune check points, PD-L1, cancer progression

## Abstract

**Simple Summary:**

Obesity is a risk factor for cancer, including breast cancer (BC). Our study proposes a novel mechanism by which it could contribute to BC progression due to interactions between mesenchymal stem cells from obese adipose tissue (ob-ASC) with infiltrating immune cells and the promotion of pathogenic cells double-secreting IL-17/IFNγ. Indeed, we demonstrated herein that the inflammatory environment mediated by the interaction of MSCs with immune cells enhanced (i) pro-inflammatory cytokine and neo-angiogenic factor secretion, (ii) metalloproteinase and (iii) immune checkpoint (ICP) over-expression, and (iv) cell migration in human breast cancer cells lines (BCCL). Moreover, (v) using neutralizing antibodies, we demonstrated the differential effects of IL-17A or IFNγ on BCCL pro-inflammatory cytokine over-expression or ICP upregulation, respectively, and the potentiating effects on BCCL migration. Finally, (vi) ICP overexpression was likely to depend on the obese status of MSCs. Therefore, our results suggest that the activation of pathogenic Th17 cells by ob-ASC could contribute to BC aggressiveness.

**Abstract:**

Background: Obesity is a well-known risk factor for cancer. We have previously reported the role of adipose-tissue-derived mesenchymal stem cells from obese individuals (ob-ASC) in the promotion of pathogenic Th17 cells and immune check point (ICP) upregulation. Thus, we postulated herein that this mechanism could contribute to breast cancer (BC) aggressiveness. Methods: Conditioning medium (CM) from mitogen-activated ob-ASC and immune cell co-cultures were added to two human breast cancer cell line (BCCL) cultures. Expressions of pro-inflammatory cytokines, angiogenesis markers, metalloproteinases, and PD-L1 (a major ICP) were measured at the mRNA and/or protein levels. BCCL migration was explored in wound healing assays. Anti-cytokine neutralizing antibodies (Ab) were added to co-cultures. Results: CM from ob-ASC/MNC co-cultures increased IL-1β, IL-8, IL-6, VEGF-A, MMP-9, and PD-L1 expressions in both BCCLs and accelerated their migration. The use of Abs demonstrated differential effects for IL-17A and IFNγ on BCCL pro-inflammatory cytokine over-expression or PD-L1 upregulation, respectively, but potentiating effects on BCCL migration. Finally, co-cultures with ob-ASC, but not lean ASC, enhanced PD-L1 expression. Conclusions: Our results demonstrate increased inflammation and ICP markers and accelerated BCCL migration following the activation of pathogenic Th17 cells by ob-ASC, which could represent a new mechanism linking obesity with BC progression.

## 1. Background

The increased incidence of obesity and its subsequent comorbidities is reaching epidemic proportions, which poses a major challenge to healthcare systems. Defined by a body mass index (BMI) > 30 kg/m^2^, obesity is due to an energy imbalance that favors weight gain and fat accumulation in adipose tissue (AT), causing metabolic disturbances and low-grade inflammation. This ultimately leads to obesity-related metabolic and/or inflammatory diseases such as cardiovascular diseases (CVD) and/or type 2 diabetes (T2D) and aggravates chronic inflammatory or auto-immune diseases as well as various cancers, including liver, colon, pancreas, prostate, and breast cancer [1,2]. Breast cancer (BC) is the most frequently diagnosed cancer in women, with 2.26 million new cases in 2019 and nearly 685,000 deaths worldwide [3]. Compared to lean women, women with obesity develop more aggressive forms of breast cancer with larger tumors, enhanced risks of metastasis and a worse response to therapies [4], which contributes to poorer prognosis and higher mortality [5,6]. Intra-tumoral inflammation and, notably, IFNγ secretion are known to impair the anti-tumoral activity of cytotoxic lymphocytes (CTL) by promoting tumor immune escape through the induction of ICP over-expression, which causes immune cell exhaustion and anergy [7,8]. Thus, because IFNγ is mainly produced by CD8+ T cells and NK cells, this allows for inhibitory feedback from tumor cells. The PD-1/PD-L1 axis is the most potent immune cell exhaustion inducer with Programmed Death-Ligand 1 (PD-L1) expressed by cancer cells and Programmed cell Death protein 1 (PD-1) by immune cells. PD-L1 inhibits both T-cell proliferation and cytotoxic function following binding to its receptor, PD-1 [9,10]. Thus, the inefficiency of the immune system in eliminating tumor cells is one of the hallmarks of cancer progression [11]. Blockade of the PD-1/PD-L1 pathway using specific antibodies has proven efficacy in immunotherapy protocols for various cancers [12,13], particularly in the treatment of metastatic triple-negative breast cancers (TNBC), which are very aggressive, with high rates of relapse and low survival rates [14,15].

Obesity-mediated inflammation results from the disruption of adipose tissue (AT) homeostasis by several mechanisms, such as pro-inflammatory adipokine secretion and/or infiltration of inflammatory immune cells from blood [16]. However, even AT from lean individuals is infiltrated by immune cells, but those ones are either regulatory T cells and/or M2 anti-inflammatory macrophages. In contrast, obese AT is infiltrated by pro-inflammatory immune cells homing there to restore AT homeostasis [17]. Indeed, to accommodate the influx of energy, AT expands in size (hypertrophy), creating a local hypoxic stress, which activates several biochemical and molecular stress pathways leading in turn to proliferation of pre-adipocytes (hyperplasia), neo-angiogenesis, and leukocyte infiltration [18]. The CD8+ T cells are the first immune cells infiltrating AT in order to lyse hypertrophic adipocytes. They then recruit M1 pro-inflammatory macrophages at their proximity in order to engulf dying cells or cellular debris. Thus, crown-like structures (CLS) with dying adipocytes surrounded by CD8+ T cells and M1 macrophages are pathognomonic of AT inflammation [19]. In addition, contributing to this obese AT sub-inflammatory state, the secretion of pro-inflammatory adipokines, such as leptin, IL1β, IL-6, TNFα, IFNγ, and IL-17A, is over-induced [17].

In a previous report, we have demonstrated the crucial role of adipose-tissue-derived mesenchymal stem cells (ASC) from obese AT (ob-ASC) in the initiation of AT inflammation, through the promotion of pathogenic Th17 lymphocytes secreting both IL17-A and IFNγ [20]. This was demonstrated using a co-culture model with ob-ASC and mitogen-activated immune mononuclear cells (MNC). Of interest, we have then shown that ob-ASC-mediated IFNγ secretion contributes to PD-L1 upregulation in (i) immune cells, (ii) ob-ASC, and (iii) bystander cells, such as mature adipocytes [21]. Thus, because AT can be found in close contact with cancer cells, such as in breast or digestive cancers, we postulated herein that one of the mechanisms linking obesity with increased risks of cancer progression could result from the AT-inflammatory environment, and more particularly from ob-ASC-mediated pathogenic Th17 activation, with (i) IFNγ contributing to PD-L1 overexpression in surrounding tumor cells and (ii) IL-17A increasing cancer cell pro-inflammatory cytokine secretion and tumorigenicity, as previously reported [8,22].

## 2. Materials and Methods

### 2.1. Isolation and Expansion of Adipose-Tissue-Derived Mesenchymal Stem Cells

Subcutaneous or visceral AT samples from 3 female subjects with obesity (BMI > 30 kg/m^2^) were obtained from residues of bariatric surgery or from residues of visceral surgery in 3 lean female subjects (BMI < 25 kg/m^2^), with the approval of the Committee for the Protection of Human Subjects of the “Hospices Civils de Lyon” and with patient’s consent. Because this study was performed on ASC from only 3 subjects, we did not blind subjects or investigators and we did not randomize subjects into groups. ASCs were isolated from AT by enzymatic digestion dissociating the stromal vascular fraction from the adipocyte fraction and were then selectively amplified in culture medium as previously reported [20]. Basal culture medium was composed of DMEM High Glucose (4.5 g/L) and Ham’s F12 1:1 supplemented with 10% Hyclone FCS, 1% glutamine, and 1% penicillin–streptomycin. ASCs were identified by immunophenotypic criteria, as recommended by the French Cell Therapy using the membrane expression of CD73, CD90, and CD105 [23] and by their ability to differentiate into cells of another origin, such as osteoblasts when cultured in an osteoblast-differentiating medium, besides differentiation into adipocytes. Calcified bone matrix was visualized by alizarin-red staining (Supplementary Figure S1).

### 2.2. Isolation of Blood Mononuclear Cells (MNC)

Blood was collected from healthy donors from the Blood Bank Center of Lyon. MNCs were isolated by Histopaque Ficoll gradient density (Sigma Aldrich, St. Louis, MO, USA) and stored in liquid nitrogen at a density of 20 × 10^6^ per mL in freezing medium (RPMI 1640 medium supplemented with 10% FCS and 10% DMSO).

### 2.3. Co-Culture Assays

In co-culture experiments, 3 distinct ASCs were plated in 12-well plates (0.2 × 10^6^ cells/well) for 24 h in RPMI supplemented with 10% FCS. Twenty-four hours later, MNCs from a single blood donor were added to ASCs, followed or not by 5μg/mL phytohemagglutinin (PHA-P), also named lectin from phaseolus vulgaris (Sigma-Aldrich, Cat#L8754), at a ratio of 5:1 (MNC:ASC), according to our previous work [20]. Three controls were used: MNCs without activation (negative control), PHA-activated MNCs without ASCs (Th1 cell activation control), and MNCs co-cultured with ASC in the absence of PHA (co-culture control). Conditioned media (CM) were collected following 48 h of culture. In blocking experiments, MNC cultures and/or ASC/MNC co-cultures were treated with anti-IL-17A (Thermo Fisher Scientific, Cat#MA5-41907, Waltham, MA, USA), anti-IFNg (Thermo Fisher Scientific, Cat#16-7318-81), or anti-TNFa (Thermo Fisher Scientific, Dardilly, France, Cat# MA5-41776) neutralizing monoclonal antibodies (mAb), each at 50 μg/mL, a concentration that has proven effectiveness at totally inhibiting the presence of these cytokines in CM.

### 2.4. Flow Cytometry Analysis

Following labelling with CD274 fluorochrome-conjugated Ab (Thermofisher, Cat# 12-5982-82), or its isotypic control as negative controls, cells were gated on their forward and side scatter properties with the exclusion of debris and dead cells. Events within gates were then analyzed for specific expression of relevant molecules, as compared to the corresponding isotypic controls. Therefore, either overlays or markers were used to identify the positive cells.

In the case of intra-cellular staining for the identification of pathogenic Th17 cells, following activation of the cells with PHA and blocking with 3.6 mmol/L brefeldin-A (GolgiPlug; Becton-Dickinson) for 4 h, the cell surface molecule of interest, such as the CD4 molecule, was first labeled with a specific fluorochrome-conjugated Ab Immunotool (Cat#21278045) or its isotypic control before permeabilization of the cells using the Cytofix/Cytoperm kit (Becton-Dickinson, Le Pont de Claix, France, Cat#54723). Then, isotypic controls corresponding to IL-17 and IFNg fluorochrome-conjugated Abs (FITC-anti IL-17 eBiosciences, Cat#11-7179-71, or PE-conjugated anti-IFNγ, (Becton-Dickinson, Le Pont de Claix, France, Cat#554701), respectively, were added to the permeabilized cells. Thirty minutes of incubation with fluorescent labelled antibodies was followed by several washes in PBS 2% FCS.

In the case of Ki-67 intra-cellular staining, the same fixation/permeabilization kit was used to permeabilize cells, and phycoerythrin-conjugated anti-Ki67 (Thermo Fisher scientific, Dardilly, France, Cat#12-5698-82) or its isotypic control were used, according to manufacturer’s instruction.

Analyses were performed as described for unpermeabilized cells using gates to exclude debris and dead cells. The LSR II cytofluorometer and the FCS express software were used.

### 2.5. Human Breast Cancer Cell Line (BCCL) Cultures

Human breast cancer cell lines (BCCL) used were MCF-7 (ATCC, Cat#HTB-22) and MDA-MB-231 (ATCC, Cat#CRM-HTB-26). Whereas the MCF-7 cell line is known to express receptors for estrogen and progesterone [24], the MDA-MB-231 cell line is triple-negative for these 2 receptors and for the human epidermal growth factor [25]. MCF-7 cells were cultured in culture medium composed of DMEM low glucose (1 g/L) supplemented with 10% FBS, 1% glutamine, 1% penicillin–streptomycin, and 0.01 mg/mL insulin, while MDA-MB-231 cells were cultured in culture medium composed of DMEM high glucose (4.5 g/L), supplemented with 10% FBS, 1% glutamine, 1% penicillin–streptomycin, and 1% non-essential amino acids.

### 2.6. Culture of Human BCCL with CM Harvested from PHA-Activated ASC/MNC Co-Cultures

MCF-7 and MDA-MB-231 cells were seeded in 24-well plates at 100,000 cells/well in their respective basal culture medium until reaching 80% confluence. Then, CM harvested from PHA-activated ASC/MNC co-cultures were added to breast cancer cell cultures. To this end, CM was diluted by half with RPMI 1640 medium, supplemented with 10% FCS. After 24 h incubation, the supernatant was harvested and frozen at −20 °C before use in ELISA for cytokine secretion measurement. Cells were collected to measure (i) mRNA levels of PD-L1, pro-inflammatory cytokines, VEGFA, and MMP9; (ii) migration; and (iii) proliferation.

### 2.7. mRNA Measurements

Total RNA was extracted from human BCCL cultures using the Tri Isolation Reagent TM (Roche Diagnostics, Meylan, France). Following lysis, total RNA was purified in 100 µL chloroform and precipitated in 100 µL isopropanol solution. Quality and quantity of total mRNA were measured using the Nanodrop 2000 (Thermo Fischer Scientific). cDNA was synthesized from 500 ng of total RNA using the Primescript-RT reverse transcription kit (Takara, Dalian, China). Quantitative RT-PCR was performed on a Rotor-Gene Real Time PCR system using the ABsolute QPCR SYBRGreen Mix (Abgene, Illkirch, France). mRNA expression levels were defined by threshold cycle (Ct) normalized to the housekeeping gene peptidyl-prolyl cis-trans isomerase (PPIF) using the mathematical method depending on ΔCT and the amplification efficiency of the transcripts, as described by Plaffl et al. [26]. The individual primer sequences used for RT-qPCR are provided in Supplementary Table S1.

### 2.8. Cytokine Secretion

Following culture with CM for 24 h at 37 °C, BCCL secretion of IL-8 (Thermo Fisher Scientific Cat#29-8089-65), IL-1β (Thermo Fisher Scientific, Dardilly, France, Cat#88-7261-88), IL-6 (Thermo Fisher Scientific, Cat#88-7066-88) and VEGF-A (Bio-Techne/R&D systems/, Noyal, Chatillon Sur Seiche, France, Cat#DY293) were measured by ELISA, using the manufacturer’s protocols. As control, CM were placed in the incubator at 37 °C for 24 h without BCCL, and residual cytokine levels were subtracted from those measured in the presence of BCCL.

### 2.9. PD-L1 Cell Surface Expression Measurements

Cell surface expression of PD-L1 in cancer cells was measured by flow cytometry as detailed above, following 24 h culture with relevant CM. BCCLs were washed in phosphate-buffered saline (PBS) and re-suspended in 100 µL staining buffer (PBS with 2% FCS). Phycoerythrin (PE) directly conjugated with anti-human PD-L1 antibodies (Thermo Fisher Scientific, Cat#12-5982-82) were used to stain BCCLs.

### 2.10. Proliferation Measurements

Ki67 labelling was used to evaluate proliferation. BCCLs (0.3 × 10^5^ cells/well) were plated in 96-well plates and incubated with recombinant human IL-17A (50 ng/mL), TNFα (50 ng/mL), IFNγ (50 ng/mL), or CM harvested from PHA-activated ASC/MNC co-cultures diluted by half with complete medium.

### 2.11. Wound Healing Assays

Wound healing assays were used to investigate cell migration. BCCLs (0.2 × 10^6^ cells/well) were plated in 24-well plates for 48 h to achieve 80% confluence. Then, a wound was formed by scratching with a pipette tip. Cells were incubated with recombinant human IL-17A (50 ng/mL), TNFα (50 ng/mL), IFNγ (50 ng/mL), or CM harvested from PHA-activated ASC/MNC co-cultures or control CM diluted by half with complete medium. BCCLs’ cell migration was measured by taking images at the beginning of cultures and 24 h later. Wound areas before and after 24 h culture were then measured and compared using the Image J Software, which allowed the calculation of % recovery.

### 2.12. Statistical Analyses

Statistical analyses were performed using the Graphpad Prims 8.4.2 software. The one-way ANOVA assay followed by post-hoc Fisher multiple comparison tests was used. ***, **, * represent significant *p* values < 0.001, <0.01, and <0.05, respectively. Data are presented as mean ± standard deviations (SD).

## 3. Results

### 3.1. Ob-ASCs Enhance IL17 and IFNγ Secretion but Inhibit TNFα Secretion in Co-Cultured MNCs

**Figure 1:**
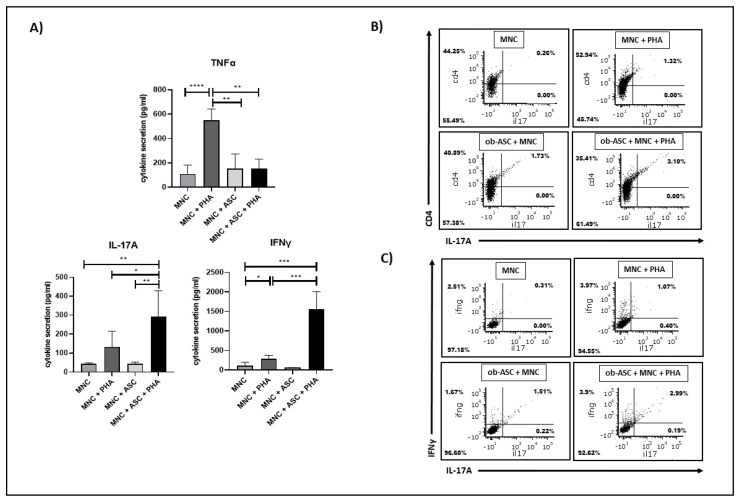
Increase of IL-17A and IFNy but decrease in TNFα, among PHA-activated ob-ASC/MNC co-cultures. MNCs from healthy donors were activated or not with PHA (5 µg/mL) and co-cultured with ob-ASCs at the MNC/ob-ASC ratio of 5:1. (**A**) After 48 h, cytokine secretion was analyzed using ELISA for TNFα, IL-17A, and IFNγ secretion. (**B**) Representative flow cytometry plots of ob-ASC/MNC stained for CD4^+^ (*y*-axis) and intracellular IL-17A^+^ (*x*-axis). The percentage of CD4^+^/IL-17A^+^ double-positive cells is indicated in the top-right quadrant. (**C**) Representative flow cytometry plots of ob-ASC/MNC stained for intracellular IFNγ^+^ (*y*-axis) and IL-17A^+^ (*x*-axis). The percentage of double-positive cells is indicated in the top-right quadrant. The percentage of IFNγ^+^/IL-17A^−^ cells is indicated in the top-left quadrant. *, **, ***, **** represent *p* < 0.05, *p* < 0.01, *p* < 0.001, *p* < 0.0001, respectively, as obtained by the one way ANOVA followed by Fisher LSD multiple comparison tests.

The co-culture model developed by our team, which mimics the infiltration of immune cells within AT from obese individuals and leads to the activation of pathogenic Th17 cells, was used [20]. To evaluate the degree of ob-ASC-mediated Th17 cell activation prior to the use of CM in BCCL cultures, we measured the levels of IL-17A and IFNγ secretion in co-cultures and of TNFα as a control of ob-ASC-mediated Th1-cell downregulation. As shown in Figure 1, following activation with PHA, a significant increase in TNFα and IFNγ secretion was observed in the MNCs, assessing for Th1 cell activation. However, while TNFα decreased during ob-ASC/MNC co-cultures, as expected for Th-1 cell downregulation, IFNγ secretion significantly increased in PHA-activated co-cultured cells together with IL-17A due to the activation of pathogenic Th17 cells, as previously reported [20] and as shown in Figure 1B,C, where IL-17 secretion was induced simultaneously with IFNg in CD4+ T cells but not in other cells in the presence of CM from PHA-activated co-cultures

### 3.2. Conditioned Medium from PHA-Activated ob-ASC/MNC Co-Cultures Enhances mRNA Expression and/or Protein Secretion Levels of Pro-Inflammatory Cytokines, VEGF-A, and MMP-9 in Human BCCLs

**Figure 2:**
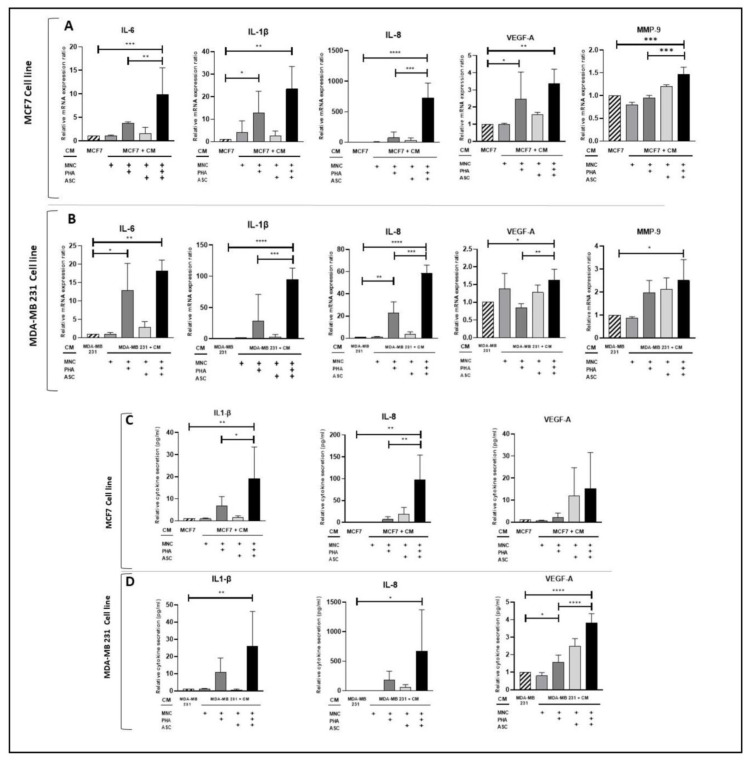
CM from PHA-activated ob-ASC/MNC co-cultures upregulates transcription and/or secretion of pro-inflammatory cytokines, VEGF-A, and MMP-9 in BCCLs. MCF7 and MDA-MB-231 (1 × 10^5^ cells/well) were cultured for 24 h with or without CM harvested from MNC cultures or ob-ASC/MNC co-cultures, which were activated or not with PHA. mRNA expression levels were measured using qRT-PCR (**A**,**B**). Cell culture supernatants were analyzed using ELISA (**C**,**D**). Results were normalized to PPIF and expressed relatively to BCCL mRNA expression or cytokine secretion. Error bars represent standard deviations (SD) from n = 3 independent experiments. *, **, ***, **** represent *p* < 0.05, *p* < 0.01, *p* < 0.001, *p* < 0.0001 respectively, as obtained by the one-way ANOVA, followed by Fisher LSD multiple comparison tests.

We then asked whether the conditioned medium (CM) collected from PHA-activated ob-ASC/MNC co-cultures could enhance BCCL inflammatory activity. To this aim, mRNA expressions of (i) pro-inflammatory cytokines, such as IL-1β, IL-8, and IL-6; (ii) VEGF-A, a pro-angiogenic factor; and (iii) MMP-9, a matrix metalloproteinase involved in breast cancer invasiveness [27], were measured in MCF-7 and MDA-MB231 cells, following 24 h culture in the presence of CM collected from PHA-activated ob-ASC/MNC co-cultures. As shown in Figure 2A,B, culture with CM harvested from PHA-activated ob-ASC/MNC co-cultures resulted in increasing IL-1β, IL-8, IL-6, VEGF-A, and MMP-9 mRNA expression levels in each BCCL, as compared to cultures without CM. In addition, CM harvested from PHA-activated MNC single cultures also enhanced the expression of some of these markers, such as IL-1β and VGFA mRNA levels in MCF-7 cells and IL-6 and IL-8 in MDA-MB231 cells. However, these markers were enhanced at much higher levels by the CM from PHA-activated ob-ASC/MNC co-culture, except for VEGF-A, the levels of which were almost similar to MCF-7 cells. To confirm these results at the protein level, cytokine secretion was then measured. As shown in Figure 2C,D, while the CM from PHA-activated co-cultures enhanced pro-inflammatory cytokine secretion in each BCCL, the CM from PHA-activated MNCs alone enhanced VEGFA levels in MDA-MB231 cells, but at much lower levels than those observed with the CM from co-cultures. Of note, IL-6 protein levels could not be determined due to the high levels of IL-6 still present in the negative control, corresponding to CM incubated for 24 h at 37 °C in the absence of a BCCL.

Thus, altogether, these results suggest a positive regulation of CM harvested from PHA-activated ob-ASC/MNC co-cultures on the transcription and/or secretion of pro-inflammatory cytokines, VEGF-A, and/or MMP-9 in MCF-7 and MDA-MB-231 BCCLs.

### 3.3. Conditioned Medium from PHA-Activated Ob-ASC/MNC Co-Cultures Enhances PD-L1 Expression in BCCLs

**Figure 3:**
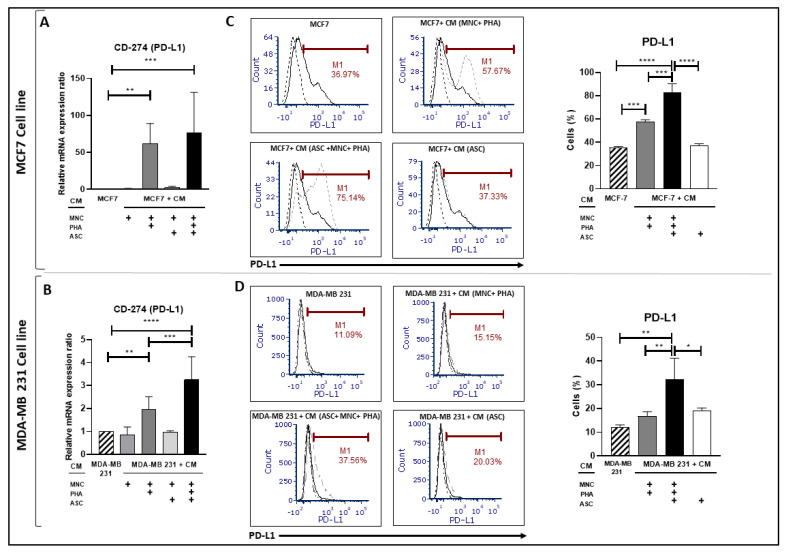
PD-L1 upregulation is mediated by PHA-activated ob-ASC/MNC co-cultures. (**A**,**B**). MCF7 and MDA-MB231 cells (1 × 10^5^ cells/well) were cultured for 24 h with or without CM harvested from MNC cultures or ob-ASC/MNC co-cultures, activated or not with PHA. mRNA expression levels were measured using qRT-PCR. Results are expressed relatively to mRNA expression in BCCLs (**C**,**D**). Cells were collected after 24 h of incubation with CM and further analyzed for PD-L1 expression at the surface membrane using flow cytometry. Histogram overlays correspond to PD-L1 expression in BCCLs cultured without CM (solid-black), versus BCCL cultured with indicated CM (solid grey). The isotypic controls are shown (hatched-black). Errors bars represent standard deviations (SD) from n = 3 independent experiments for mRNA and Flow cytometry. *, **, ***, **** represent *p* < 0.05, *p* < 0.01, *p* < 0.001, *p* < 0.0001 respectively, as obtained by the one-way ANOVA, followed by Fisher LSD multiple comparison tests.

Since inflammation mediated by ob-ASCs has been shown to (i) contribute to PD-L1 upregulation in monocytes and ob-ASC themselves and (ii) to spread towards bystander cells, such as mature adipocytes [21], we then investigated whether such spreading could occur towards nearby cancer cells. Thus, CM collected from PHA-activated ob-ASC/MNC co-cultures was added to human BCCL cultures for 24 h. This resulted in the enhancement of PDL-1 mRNA transcription at levels significantly higher than those obtained following culture with the CM from PHA-activated MNC single cultures used as a control (Figure 3A,B). These results were then confirmed at the protein level since surface membrane expression of PD-L1 increased at the highest levels in the presence of CM from PHA-activated ob-ASC/MNC co-cultures, as compared with CM from PHA-activated MNC (Figure 3C,D). Moreover, CM from MNC (Figure 3A,B) or ob-ASC (Figure 3C,D) cultures did not induce any increase in PD-L1 expression. Thus, these data suggest that ob-ASC/MNC interactions may mediate PD-L1 overexpression in tumor cells through the secretion of soluble factors.

### 3.4. PD-L1 Over-Expression Is Dependent on IFNγ, While Enhanced Pro-Inflammatory Cytokine Secretion Is Influenced by IL-17A and/or TNFα in BCCLs Cultured with CM from PHA-Activated Ob-ASC/MNC Co-Cultures

**Figure 4:**
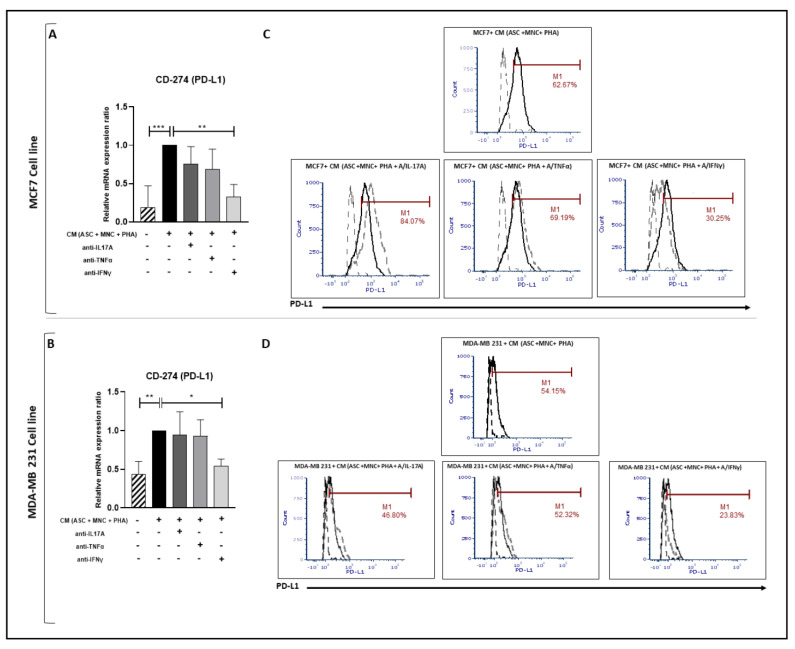
PD-L1 overexpression in BCCLs is partially inhibited by CM with IFNγ neutralizing antibodies. MCF7 and MDA-MB-231 (1 × 10^5^ cells/well) cells were cultured for 24 h with CM harvested from PHA-activated ob-ASC/MNC co-cultures (i) plus or minus A/IL-17A, (ii) A/TNFα, or (iii) A/IFNγ mAbs. (**A**,**B**). mRNA expression levels were measured using qRT-PCR. Results were normalized to PPIF and expressed relative to BCCLs cultured with CM from activated co-cultures. (**C**,**D**) PD-L1 membrane expression was measured using flow cytometry. Histogram overlays correspond to isotypic control (black and hatched) versus PD-L1 with CM (solid and black), or with CM plus mAbs (hatched and grey). *, **, *** represent *p* < 0.05, *p* < 0.01, *p* < 0.001, respectively, as obtained by the one-way ANOVA, followed by Fisher LSD multiple comparison tests.

To test whether T cell cytokines secreted during PHA-activated ob-ASC/MNC co-cultures could be involved in the enhancement of pro-inflammatory cytokine and VEGF-A secretion and in ICP upregulation, neutralizing antibodies directed against IL-17A, IFNγ, or TNFα were added during PHA-activated ob-ASC/MNC co-cultures. The collected CM were then added to BCCL cultures for 24 h. As shown in Figure 4A,B, PD-L1 mRNA overexpression in each BCCL was inhibited in the presence of anti-IFNγ but not anti-IL-17 or anti-TNFα mAbs, thus supporting the specific role of IFNγ in PD-L1 transcription, as previously reported [7,8,21]. These results were confirmed at the protein level (Figure 4C,D).

**Figure 5:**
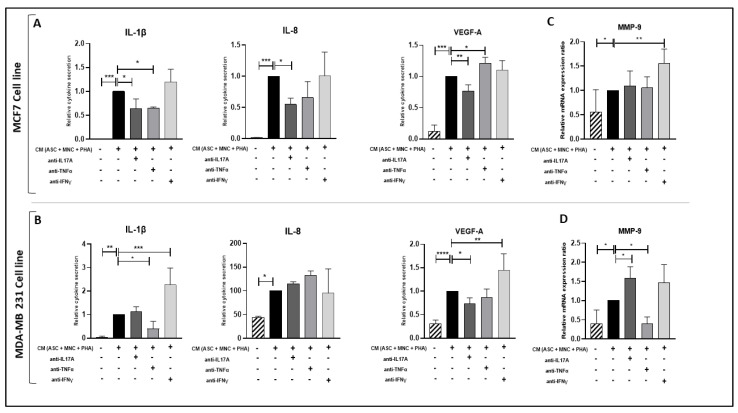
Pro-inflammatory cytokine secretion of BCCLs is partially inhibited by CM containing IL-17A or TNFα neutralizing antibodies. MCF7 and MDA-MB-231 cells (1 × 10^5^ cells/well) were cultured for 24 h with CM harvested from PHA-activated ob-ASC/MNC co-cultures plus or minus A/IL-17A, (ii) A/TNFα, or (iii) A/IFNγ mAbs. Cell culture supernatants were analyzed using ELISA (**A**,**B**), and MMP-9 mRNA expression levels were measured using qRT-PCR (**C**,**D**). Results were expressed relative to the mRNA levels of BCCLs cultured with CM from activated co-cultures. *, **, ***, **** represent *p* < 0.05, *p* < 0.01, *p* < 0.001, and *p* < 0.0001 respectively, as obtained by the one-way ANOVA, followed by Fisher LSD multiple comparison tests of 3 different experiments.

In contrast, anti-IFNγ mAb did not inhibit the increase in pro-inflammatory cytokine or VEGF-A secretion (Figure 5A,B) nor in MMP-9 transcription, but rather increased them (Figure 5C,D), suggesting either an anti-inflammatory role for IFNg or a regulatory role for other cytokines involved in such inflammation. TNFα was also likely to play a role in IL-1β cytokine secretion in both BCCL and in MMP9 expression in MDA-MB231, while IL-17A was more likely to contribute to VEGF-A over-secretion in each BCCL and in IL-1β and IL-8 over-secretion in MCF-7 cells (Figure 5B,C), as assessed by the inhibition of these factors with respect to the presence of the relevant neutralizing Abs. Overall, these results suggest that ob-ASC/MNC interactions are likely to enhance pro-inflammatory cytokine and VEGF-A secretion in BCCLs through IL-17A as well as TNFα cytokine secretion, while PD-L1 overexpression is more likely to depend on IFNγ.

### 3.5. CM from PHA-Activated Ob-ASC-MNC Co-Cultures Enhances BCCL Migration but Not Proliferation

**Figure 6:**
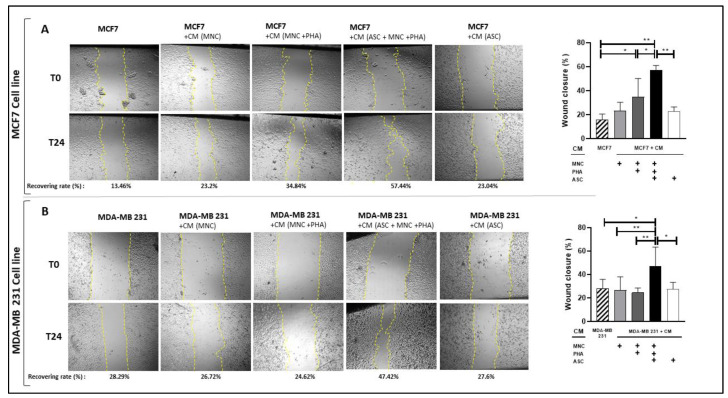
CM from PHA-activated ob-ASC/MNC co-cultures enhances BCCL migration. MCF7 (A) and MDA-MB 231 (B) (1 × 10^5^ cells/well) cells were cultured for 24 h, with or without CM harvested from MNC or ob-ASC cultures or from ob-ASC/MNC co-cultures activated or not with PHA. Cell migration was evaluated by measuring the monolayer gap closure with the help of the ImageJ software, before and after 24 h of culture. Yellow dotted lines represent wound area, relative to the initial wound area. Results are presented as % of wound recovery. Error bars represent standard deviations (SD) from n = 3 independent experiments. *, ** represent *p* < 0.05, *p* < 0.01, respectively, as obtained by the one-way ANOVA, followed by Fisher LSD multiple comparison tests.

We next measured the effects of CM from PHA-activated ob-ASC/MNC co-cultures on BCCL cell migration by using wound healing assays following the 24 h culture of BCCLs with such a CM. The effects of the CM from (i) MNC cultures activated or not with PHA or (ii) complete medium were measured in parallel as controls. As shown in Figure 6, wound recovery reached a value of 57% in the presence of CM from PHA-activated ob-ASC/MNC co-cultures, while it reached a value of 13% in the MCF-7 negative control and almost 35% with CM from the PHA-activated MNC single cultures. As a control, the CM from the MNC or ob-ASC cultures did not increase MCF-7 migration. In MDA-MB231 cells, the CM from the PHA-activated ob-ASC/MNC co-cultures induced a wound recovery of 47%, while the healing in the presence of the CM from the PHA-activated MNC single cultures was almost similar to the negative controls. To then address whether enhanced proliferation of BCCL could contribute to the enhanced wound healing that was observed in the presence of the CM from the PHA-activated ob-ASC/MNC co-cultures, we labeled MDA-MB231 cells for Ki-67. However, no increase in Ki-67 expression was observed. This was supported by the mRNA expression levels of cyclin D1, which remained stable in both cell lines regardless of whether BCCLs were cultured with the CM from the PHA-activated ob-ASC/MNC co-cultures or not (Supplementary Figure S2). Thus, altogether, these results strongly suggest that ob-ASC/MNC interactions secrete factors able to induce BCCL cell migration.

### 3.6. Pathogenic Th17 Cell Cytokines Contribute to BCCL Migration Following Culture with CM from PHA-Activated Ob-ASC/MNC Co-Cultures

**Figure 7:**
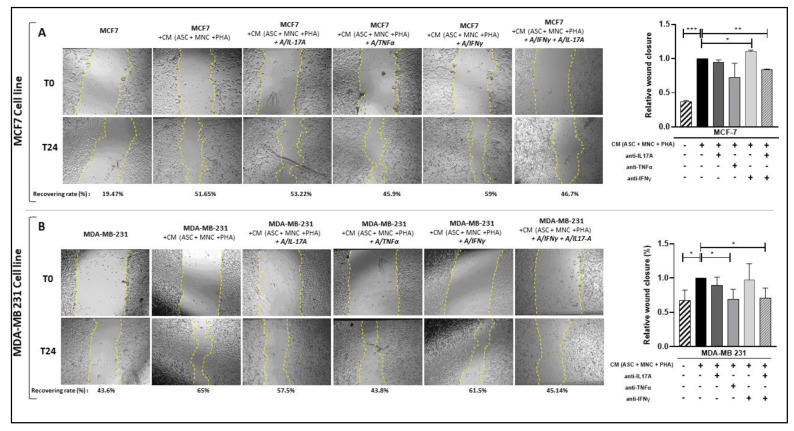
Pathogenic Th17 cell cytokines are involved in the enhancement of BCCL migration mediated by CM from PHA-activated ob-ASC/MNC co-cultures. (**A**,**B**) MCF7 and MDA-MB-231 cells were cultured for 24 h with or without CM harvested from PHA-activated ob-ASC/MNC co-cultures, plus or minus A/IL-17A, (ii) A/TNFα, (iii) A/IFN-γ mAbs, or (iv) a combination of A/IL-17A and A/IFN-γ mAbs. Cell migration was evaluated by measuring the monolayer gap closure with the help of the ImageJ software, before and after 24 h culture. Yellow dotted lines represent wound area Results are presented as % of wound recovery, relative to the initial wound area. In histograms results are expressed relatively to wound recovery of BCCLs cultured with CM from activated cocultures. Error bars represent standard deviations (SD) from n = 3 independent experiments. *, **, ***, represent *p* < 0.05, *p* < 0.01, *p* < 0.001, respectively, as obtained by the one-way ANOVA, followed by Fisher LSD multiple comparison tests.

To then determine whether the Th17 cell cytokines secreted during PHA-activated ob-ASC/MNC co-cultures could be involved in the increase in BCCL migration, we treated this CM with or without mAbs directed against IL-17A, IFNγ, or TNFα as a control of Th1 cytokines and measured their effects in wound healing assays. As shown in Figure 7, while, as expected, wound recovery was enhanced in the presence of the CM from the PHA-activated ob-ASCs/MNCs co-cultures, no inhibition of wound healing occurred in the presence of Abs against IL-17 or IFNγ. This was supported by the expression of CX3CR1, a chemokine receptor known to be involved in BC cell migration [28], which was increased in MDA-MB231 cells cultured with the CM from the PHA-activated ob-ASC/MNC co-cultures but unaffected by these Abs (Supplementary Figure S2). However, when both IL-17 and IFNg were simultaneously neutralized, a significant inhibition of wound healing was demonstrated in each BCCL.

**Figure 8:**
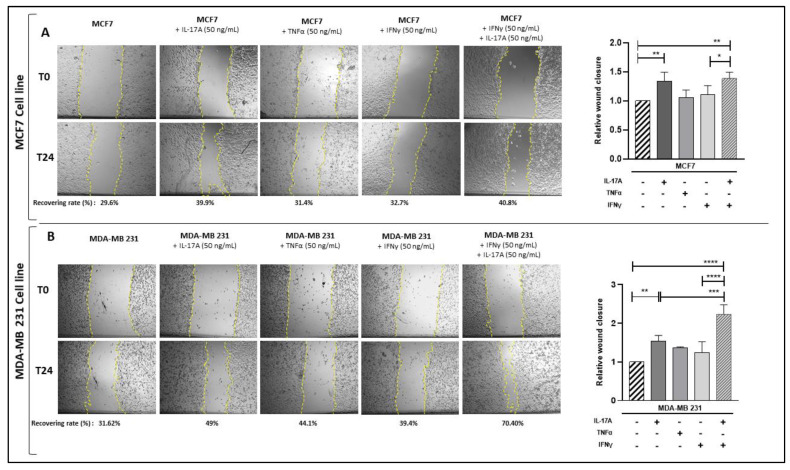
Pathogenic Th17 cell cytokines enhance BCCL migration. (**A**,**B**) MCF7 and MDA-MB-231 cells were cultured for 24 h with or without recombinant human (i) IL-17A (50 ng/mL), (ii) TNFα (50 ng/mL) (iii) IFNγ (50 ng/mL) and (iv) a combination of IL-17A (50 ng/mL) and IFNγ (50 ng/mL). Cell migration was evaluated by measuring the monolayer gap closure with the help of the ImageJ software, before and after 24 h culture. Yellow dotted lines represent wound area Results are presented as % of wound recovery, relative to the initial wound area. In histograms, results are expressed relatively to wound recovery of untreated BCCL. Error bars represent standard deviations (SD) from n = 3 independent experiments. *, **, ***, **** represent *p* < 0.05, *p* < 0.01, *p* < 0.001, *p* < 0.0001 respectively, as obtained by the one-way ANOVA, followed by Fisher LSD multiple comparison tests.

To confirm these results, we reciprocally supplemented BCCL cultures with IL-17A and/or IFNγ or TNFα and measured wound healing after 24 h. As shown in Figure 8, this resulted in a positive effect of IL-17 on BCCL migration and a potentiation by IFNγ in MDA-MB231 cells, whereas TNFα did not induce any significant migration. Therefore, altogether, these results suggest a direct effect of pathogenic Th-17 cytokines on BCCL migration.

### 3.7. Enhancement of Pro-Inflammatory Cytokine and PD-L1 mRNA Expressions in BCCL Is Mediated by Obese Rather Than Lean ASCs

**Figure 9:**
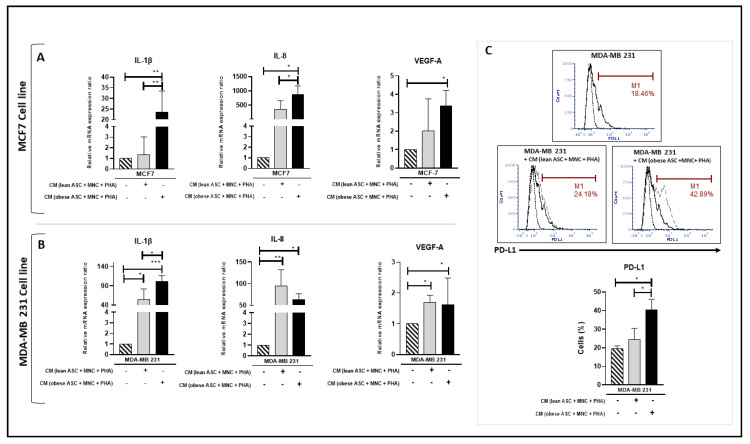
CM from obese rather than lean ASCs upregulates pro-inflammatory cytokine and PD-L1 expression. (**A**,**B**) MCF7 (**A**) and MDA-MB 231 (**B**) were cultured with CM harvested from PHA-activated lean or obese ASC/MNC co-cultures, where MNCs were from a single source, but ASCs were collected from 3 lean subjects or 3 obese subjects. mRNA expression levels were measured using qRT-PCR, and results were normalized to PPIF, relative to their expression in BCCLs. (**C**) PD-L1 expression in MDA-MB231 cells was measured using flow cytometry. Histogram overlays correspond to isotypic control (hatched-black) versus PD-L1 in BCCL (solid-black) or PD-L1 with CM (hatched-grey). Error bars represent standard deviations (SD) from n = 3 independent experiments. *, **, and ***, represent *p* < 0.015, *p* < 0.01, and *p* < 0.001, respectively, as obtained by the one-way ANOVA, followed by Fisher LSD multiple comparison tests.

To then investigate the impact of obesity in this model, we compared the effects of CM collected from obese versus lean ASC/MNC interactions on pro-inflammatory cytokine and PD-L1 mRNA expressions in BCCLs. As shown in Figure 9A,B, we observed that the CM from lean versus obese ASCs’ interactions with MNCs was unable to significantly enhance IL-1β, IL-8, and/or VEGFA mRNA expression levels in MCF-7 cells. However, in the case of MDA-MB 231 cells, IL-8 levels were unexpectedly higher in the presence of the CM from lean as compared to obese ASC/MNC interactions. Furthermore, IL-1β transcription was induced at higher levels with obese as compared to lean ASCs. Finally, PD-L1 cell surface expression was enhanced with the CM from obese but not lean ASC/MNC co-cultures (Figure 9C).

Thus, altogether, these results suggest the upregulating effects of obese versus lean ASC/MNC interactions on PD-L1 transcription in BC cells.

## 4. Discussion

Cancer progression can be promoted by inflammatory and/or pro-angiogenic factors present in the tumor microenvironment but can also result from immune escape [29]. Indeed, over time, the tumor environment becomes immunosuppressive with increased frequency of regulatory T cells, type 2 macrophages, and myeloid-derived suppressor cells. However, cancer cells themselves can also contribute to immune escape by overexpressing molecules named immune checkpoints (ICP), which are known to regulate exacerbated immune responses [9,10]. Among them, the PD-1/PD-L1 axis is the strongest exhaustion inducer of the immune system. It is transiently overexpressed following T cell activation but can also be robustly and durably overexpressed in cancer cells which exploit this mechanism to inhibit immune responses, notably the anti-tumor cytolytic activities of CD8+ T cells and/or natural killer cells [30,31]. Importantly, a recent study has demonstrated that T cells from tumor-bearing obese mice exhibited increased exhaustion compared to T cells from tumor-bearing lean mice, which was correlated with increased PD-1 expression [32]. Supporting the impact of obesity on ICP overexpression, we have previously reported enhanced PD-1 and PD-L1 expression in the AT of obese mice compared to lean mice [21]. Using an in vitro model mimicking AT inflammation in obese individuals and leading to enhanced secretion of pro-inflammatory factors, we have indeed shown that PHA-activated ob-ASC/MNC co-cultures resulted in upregulated ICP expression among interacting cells, which spread towards bystander cells [21]. Because AT and epithelial cells, including malignant cells, are found in close proximity in BC, where fat provides a source of energy, as well as a variety of soluble factors, to tumor cells [33], we asked herein whether ob-ASC/MNC interactions could also induce ICP upregulation and propagate inflammation towards nearby cancer cells. To answer this question, the effects of CM from PHA-activated ob-ASC/MNC co-cultures were tested on two different human BCCLs, MCF7 and MDA-MB231, which express hormone and EGF receptors or not, respectively. Interestingly, we observed that the CM harvested from the PHA-activated ob-ASC/MNC co-cultures was able to propagate inflammation towards each BCCL, as assessed by increased transcription and/or secretion levels of IL-1β, IL-6, and IL-8 pro-inflammatory cytokines and of the pro-angiogenic factor VEGF-A (Figure 2). In addition to the enhancement of IL-1β, IL-6, and IL-8 transcription, we observed an increase in MMP-9 mRNA levels. Accordingly, IL-1β is known to increase BC aggressiveness [34] and plays a key role in the initiation of the metastatic process by increasing MMP-9 and VEGFR expression through increased activation of p38-MAPK and MAPK-activated protein kinase 2, leading to endothelial cell migration and tumor progression [35]. Moreover, IL-8 and IL-6 have been associated with increased invasiveness metastatic potential and cancer recurrence in breast cancer cells [36,37]. The control CM from cultured ob-ASCs did not demonstrate any effect on cancer cell cytokine secretion, except for a moderate increase in IL-8 in one BCCL, which was 100 times less than that induced by the CM from the activated co-cultures (Supplementary Figure S4). In addition to the amplification of a pro-inflammatory profile, we showed that the CM from the PHA-activated ob-ASC/MNC co-cultures was also able to increase the migration of MCF-7 and MDA-MB231 cells. Indeed, wound healing increased almost twice after 24 h of culture with this CM compared to cultures in the absence of any CM or with CM collected from PHA-activated MNC single cultures (Figure 6). This was unlikely to result from increased proliferation, as assessed by Ki67 staining and Cyclin D expression, which remained unchanged whether the CM from the PHA-activated ob-ASC/MNC co-cultures was added or not (Supplementary Figure S2). Interestingly, the concomitant neutralization of IL17 and IFNg inhibited wound healing (Figure 7), which demonstrated the contribution of pathogenic Th17 cytokines in BCCL migration. Supporting these results, the direct supplementation of IL-17 in BCCL cultures led to an increase in BCCL migration, which was potentiated by IFNg (Figure 8). Whereas the neutralization of TNFa inhibited only CM-mediated MDA-MB231 migration, no direct effects of TNFa on BCCL migration was observed, suggesting an indirect effect of this cytokine. Because no increase (or compensation) in IL-17 or IFNg was observed upon TNFa neutralization, as shown in Supplementary Figure S5, this suggests the involvement of other over-secreted inflammatory cytokines, possibly IL-6 or IL-8, as reported by others [36,37].

We then investigated whether ob-ASC/MNC interactions could help BC cells escape from anti-tumoral immune responses by measuring PD-L1 expression. The data shown in Figure 3 indeed demonstrate a strong upregulation of PD-L1 expression at both the mRNA and protein levels in each BCCL, thus supporting the positive impact of obesity on cancer cell immune escape [32]. Moreover, this effect was likely to depend on the obese state of ASCs, since culture with the CM from the lean ASC/MNC co-cultures did not upregulate PD-L1 expression (Figure 8). Some soluble factors, which preponderantly derive from immune cells, have been reported to contribute to PD-L1 over-expression, such as IL-17, TNFα, and/or IFNγ, with IFNγ being the most robust one [8,38,39]. By adding specific neutralizing antibodies to these cytokines during PHA-activated ob-ASC/MNC cultures, our data only supported the influence of IFNγ on PD-L1 overexpression in BCCLs (Figure 4). IL-17 was rather involved in the upregulation of pro-inflammatory cytokines and VEGF-A secretion in BCCLs (Figure 5). Accordingly, IL-17A/F secreted by pathogenic Th17 cells has been shown to contribute to the growth and metastasis of numerous cancers via increased secretion of IL-6 by tumor cells and tumor-stromal cells, resulting in Stat-3 activation [22]. Thus, even though differential effects of pathogenic Th17-cell cytokines were observed, their concomitant action was demonstrated to promote BCCL migration.

## 5. Conclusions

ASCs, notably when issued from obese individuals, have already been described as important players in tumor progression by (i) increasing neo-vascularization, (ii) differentiating into adipocytes to provide a source of energy, (iii) enhancing cancer growth and invasion, (iv) inducing epidermal–mesenchymal cell transition, and/or (v) increasing resistance to chemotherapy [40,41,42,43,44]. However, our present data suggest a novel mechanism by which ob-ASCs could contribute to BC progression. This may result from the interaction of ob-ASC with AT-infiltrating immune cells, which leads to the activation of pathogenic Th17 cells double secreting IL-17 and IFNγ and able to increase pro-inflammatory cytokine and neo-angiogenic factor secretion in tumor cells and stimulate ICP over-expression. Moreover, the well-known enhanced ability of ASCs from obese individuals to migrate towards tumors [45], and thus to encounter tumor-infiltrating immune cells, leads us to suggest that such a mechanism could be present in other types of cancer. Blocking physical interactions between ob-ASCs and immune cells could be a new way to prevent in situ inflammation and cancer progression. We indeed have previously demonstrated that ICAM-1 partly contributes to this interaction and to subsequent Th17 cell polarization [20,46]. However, due to the systemic role of ICAM-1, other molecules acting more specifically on ob-ASC and immune cell contacts are under investigation.

## 6. Limits of the Study

The overexpression of PD-L1 is known to induce intrinsic effects in cancer cells, such as the epithelial–mesenchymal transition (EMT) [47,48]. While ob-ASCs are also known to activate EMT [42], this issue could not be addressed herein but deserves further investigation to better understand whether ob-ASCs-mediated EMT transition could be related to the induction of PD-L1 overexpression or not. Moreover, the development of organoids should help to measure the effects of PD-L1 overexpression on cancer cell progression, notably on migration, putative EMT transition, and/or inhibition of anti-tumor immune responses.

## Data Availability

The datasets used and/or analyzed in the current study are available from the corresponding author, on reasonable request.

## Supplementary Materials

The following supporting informations can be downloaded at: https://www.mdpi.com/article/10.3390/cancers15112963/s1, Figure S1: Characterization of Adipose tissue-derived-mesenchymal stem cells (ASC). ASC were stained for CD90, CD73 and CD105. The expression of these surface molecules was measured by flow cytometry. This figure is representative of 3 experiments. (B) ASC were differentiated or not (as control) into osteoblasts using osteoblast differentiation medium for 14 days and stained with alizarin red (calcium deposits). Figure S2: CM from activated co-cultures does not influence BCCL proliferation, but migration. (A) MDA-MB-231 cells (0.3 × 10^5^ cells) were cultured for 24 h with or without CM harvested from with PHA activated ob-ASC/MNC co-cultures and Ki67 expression were measured by flow cytometry. Overlays correspond to isotypic control (hatched-black) versus Ki67 in MDA-MB231 (solid-black) or MDA-MB231 plus CM (hatched-grey). Statistical analysis of 3 experiments is shown. (B) CyclinD1 mRNA expression levels were measured in each BCCL by qRT-PCR and results were normalized to PPIF. (C) MDA-MB 231 were cultured with CM harvested from PHA-ob-ASC/MNC cocultures plus or minus (i) A/IL-17A, (ii) A/TNFα or (iii) A IFNγ. CX3CR1 expression levels were measured by qRT-PCR and results were normalized to PPIF. * represent *p* < 0.05 as obtained by the one way ANOVA followed by Fisher LSD multiple comparison tests Figure S3: MDA-MB 231 cell proliferation is not influenced by pathogenic Th17cell cytokines. (A) MDA-MB-231 cells (0.3 × 10^5^ cells) were cultured for 24 h with or without CM harvested from with PHA activated ob-ASC/MNC co-cultures plus or minus A/IL-17A, (ii) A/TNF- α, (iii) A/IFN-γ mAbs or (iv) combination of A/IL-17A and A/IFN-γ mAbs and Ki67 expression were measured by flow cytometry. (B) MDA-MB-231 cells (0.3 × 10^5^ cells) were cultured for 24 h with or without recombinant human (i) IL-17A (50 ng/mL), (ii) TNFα (50 ng/mL), (iii) IFN (50 ng/mL) and (iv) combination of IL-17A (50 ng/mL) and IFN (50 ng/mL) Ki67 expression were measured by flow cytometry. Overlays correspond to isotypic control (hatched-black) versus Ki67 in MDA-MB 231 (solid-black) or MDA-MB231 plus cytokines (grey). Statistical analysis of 3 experiments is shown. Figure S4: BCCL cytokine secretion profile following culture with Ob-ASC CM. MCF7 and MDA-MB-231 cells (1 × 10^5^ cells/well) cells were cultured for 24 h with CM harvested from ob-ASC alone. (A,B) Cell culture supernatants were analyzed by ELISA. Error bars represent standard deviations (SD) from n = 3 independent experiments. *, ** represent *p* < 0.05, *p* < 0.01, respectively, as obtained by the one-way ANOVA, followed by Fisher LSD multiple comparison tests. Figure S5: Neutralization of T cell cytokines in ob-ASC/MNC cocultures and residual cytokine levels among CM. MNC from healthy donors were activated or not with PHA (5 µg/mL) and co-cultured with ob-ASC (5:1 ratio) (i) plus or minus A/IL-17A, (ii) A/TNFα or (iii) A/IFNγ) mAbs. 48 h later, cytokine secretion was analyzed by ELISA for TNFα, IL-17A and IFNγ secretion. *, **, ***, **** represent *p* < 0.05, *p* < 0.01, *p* < 0.001, *p* < 0.0001, respectively, as obtained by the one way ANOVA followed by Fisher LSD multiple comparison tests. Table S1: Primers for Human transcripts used in real-time PCR.

## Abbreviations

AT: Adipose tissue; ASC: Adipose-derived mesenchymal stem cells; BC: Breast cancer; BCCL: Breast cancer cell line; BMI: Body mass index; CLS: Crown-Like structures; CM: Conditioned media; CVD: Cardiovascular diseases; CTL: Cytotoxic lymphocytes; FCS: Fetal calf serum; HER2: Human Epidermal Growth Factor Receptor-2; mABS: Monoclonal antibodies; MNC: Mononuclear cell; PD-1: Programmed cell death 1; PD-L1: Programmed death ligand 1; PE: Phycoerythrin; PHA: Phytohemagglutinin; TNBC: Triple-negative breast cancer.
