# Supplementary material for: Contribution of Mesenchymal Stem Cells from Obese Adipose Tissue to PD-L1 Over-Expression and Breast Cancer Progression through Pathogenic Th17 Cell Activation
Source: Cancers (Basel). 2023 May 29;15(11):2963. doi: 10.3390/cancers15112963 (PMC10252031; doi:10.3390/cancers15112963)
Supplement: Supplementary file 1 [file cancers-15-02963-s001.zip › cancers-2346478-supplementary.pdf]

Supplementary Material

Contribution of Mesenchymal Stem Cells from Obese Adipose Tissue to PD-L1 Over-Expression and Breast Cancer Progression through Pathogenic Th17 Cell Activation

Ferdinand Blangero [1], Maud Robert [1,2], Thomas Andraud [1], Charles Dumontet [3], Hubert Vidal [1] and Assia Eljaafari [1,4,*]

[1] CarMeN Laboratory, INSERM U1060, INRAE U1397, University Claude Bernard Lyon Hospices Civils de Lyon, Centre Hospitalier Lyon Sud, 69310 Pïerre Bénite ; France;
[2] Bariatric Surgery Department, Edouard Herriot Hospital, 69003 Lyon, France;
[3] Center of Research in Cancerology of Lyon, INSERM U1052, CNRS 5286, University Claude Bernard Lyon 1, Centre Léon Berard, 69008 Lyon, France;
[4] Research Department, Hospices Civils de Lyon,69002 Lyon; France
* Correspondence: assia.eljaafari@univ-lyon1.fr. T

Table S1. Primers for Human transcripts used in real-time PCR

Gene name	5'-3' sequence	Amplicon size, bp	RefSeq number
PPIF	F : GGCTACAAAGGCTCCACCTTC<br>R : GAAAGCGGCTTCCGTAGATG	112	NM_005729
IL-6	F : AGCCCTGAGAAAGGAGACATGTAACAAG<br>R : TTCTGCAGGAACTGGATCAGGACTTT	273	NM_000600
IL-1β	F : GGCAATGAGGATGACTTGTT<br>R : TGTAGTGGTGGTCGGAGATT	124	NM_000576
IL-8	F : TTCTGCAGCTCTGTGTGAAG<br>R : ACAGAGCTCTCTTCCATCAG	187	NM_000584
VEGF-A	F : AGTACCCTGATGAGATCGAG<br>R : TATGTGCTGGCCTTGGTGAG	161	NM_001025366
MMP-9	F : GGGAAGATGCTGCTGTTCA<br>R : TTCAACTCACTCCGGGAACT	203	NM_004994
CD-274	F : CTGCAGGGCATTCCAGAAAG<br>R : GTTCAGCAAATGCCAGTAGG	71	NM_001314029
CX3CR1	F : AGAGGCTGGTTCTTACGATG<br>R : CAGGTTGGCAGTAGCATGAA	170	NM_001171172
CyclinD1	F : CTTCCTCTCCAAAATGCCAG<br>R : AGGGCGGATTGGAAATGAAC	113	NM_053056

A

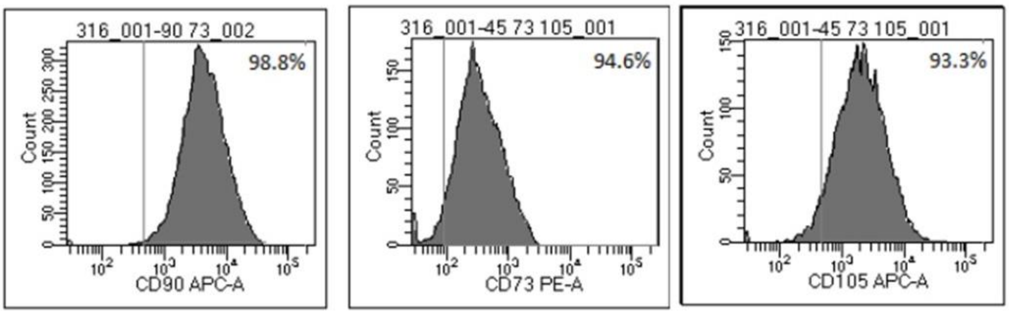

B

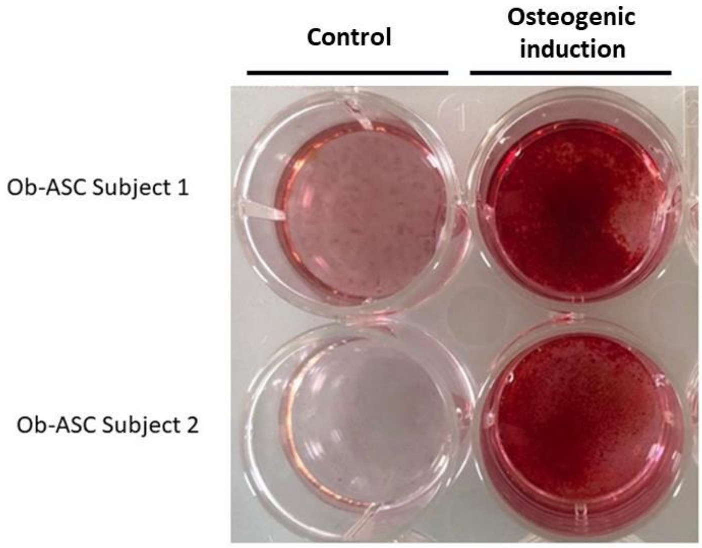

Supplementary Figure S1. Characterization of Adipose tissue-derived-mesenchymal stem cells (ASC).
(A) ASC were stained for CD90, CD73 and CD105. The expression of these surface molecules was measured by flow cytometry. This figure is representative of 3 experiments. **(B)** ASC were differentiated or not (as control) into osteoblasts using osteoblast differentiation medium for 14 days and stained with alizarin red (calcium deposits).

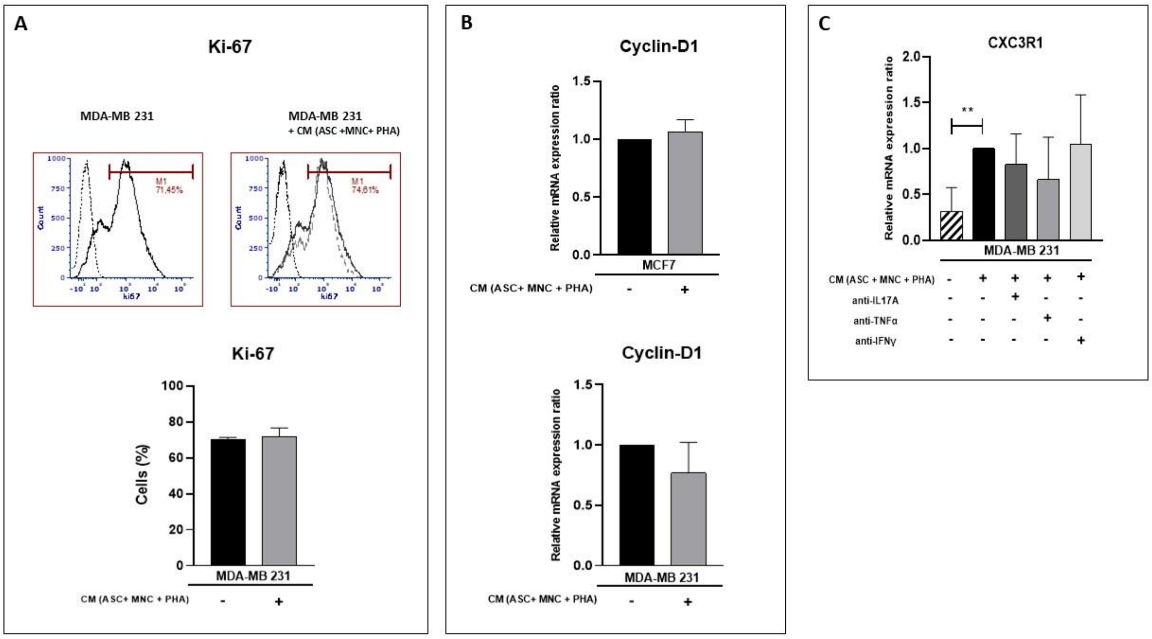

Supplementary Figure S2. CM from activated co-cultures does not influence BCCL proliferation , but migration

(A) MDA-MB-231 cells (0,3 x 10^5 cells) were cultured for 24 hours with or without CM harvested from with PHA activated ob-ASC/MNC co-cultures and Ki67 expression were measured by flow cytometry. Overlays correspond to isotypic control (hatched-black) versus Ki67 in MDA-MB231(solid-black) or MDA-MB231 plus CM (hatched-grey). Statistical analysis of 3 experiments is shown. **(B)** CyclinD1 mRNA expression levels were measured in each BCCL by qRT-PCR and results were normalized to PPIF. **(C)** MDA-MB 231 were cultured with CM harvested from PHA-ob-ASC/MNC cocultures plus or minus (i) A/IL-17A, (ii) A/ TNFα or (iii) A IFNγ. CX3CR1 expression levels were measured by qRT-PCR and results were normalized to PPIF. * represent $p < 0.05$ as obtained by the one way ANOVA followed by Fisher LSD multiple comparison tests.

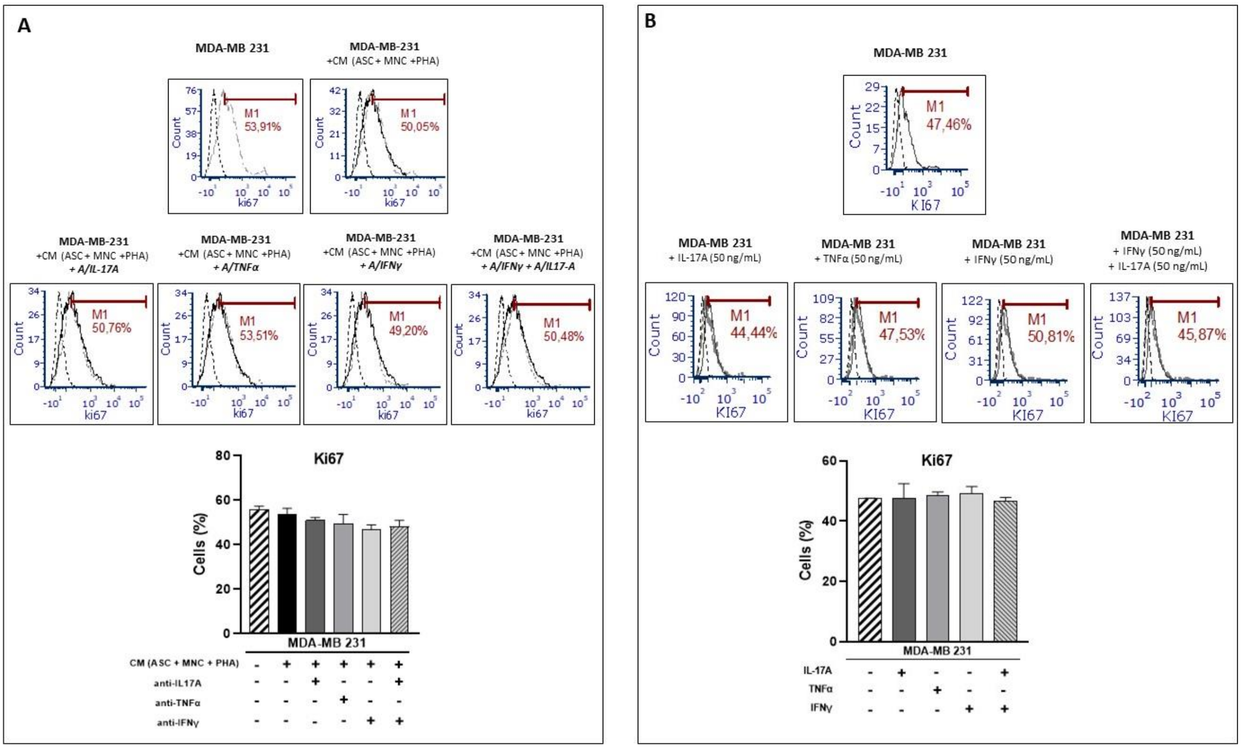

Supplementary Figure S3: MDA-MB 231 cell proliferation is not influenced by pathogenic Th17cell cytokines
(A) MDA-MB-231 cells (0,3 x 10^5 cells) were cultured for 24 hours with or without CM harvested from with PHA activated ob-ASC/MNC co-cultures plus or minus A/IL-17A, (ii) A/ TNF- α, (iii) A/ IFN-γ mAbs or (iv) combination of A/IL-17A and A/IFN-γ mAbs and Ki67 expression were measured by flow cytometry. **(B)** MDA-MB-231 cells (0,3 x 10^5 cells) were cultured for 24 hours with or without recombinant human (i)IL-17A (50 ng/mL), (ii) TNFα (50 ng/mL), (iii) IFNγ (50 ng/mL) and (iv) combination of IL-17A (50 ng/mL) and IFNγ (50 ng/mL) Ki67 expression were measured by flow cytometry. Overlays correspond to isotypic control (hatched-black) versus Ki67 in MDA-MB 231(solid-black) or MDA-MB231 plus cytokines (grey). Statistical analysis of 3 experiments is shown.

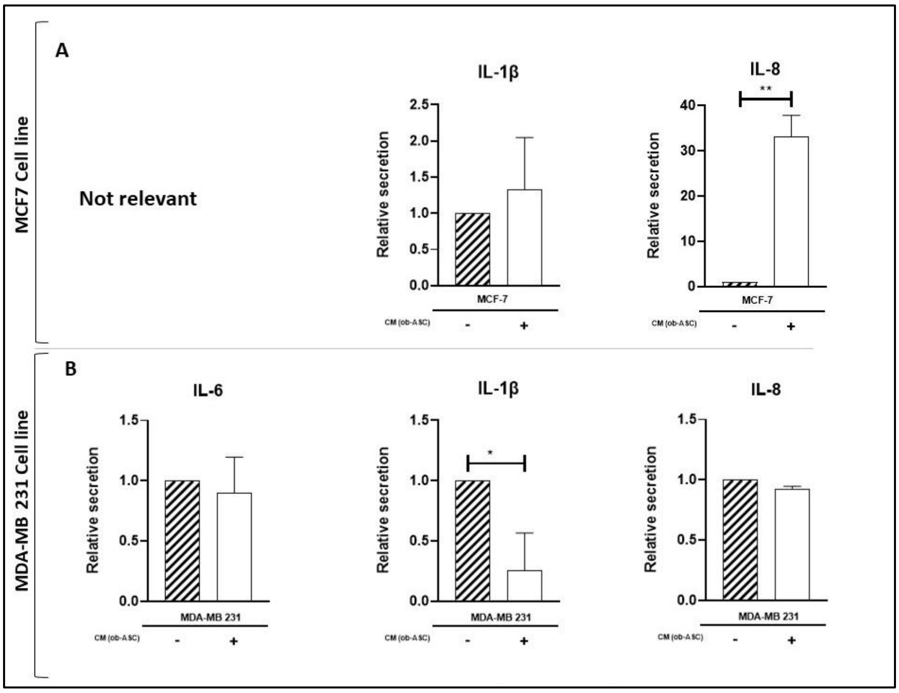

Supplementary Figure S4 : BCCL cytokine secretion profile following culture with Ob--ASC CM
MCF7 and MDA-MB-231 cells (1 x 10^5 cells/well) cells were cultured for 24h with CM harvested from ob-ASC alone. **(A,B)** Cell culture supernatants were analyzed by ELISA. Error bars represent standard deviations (SD) from n = 3 independent experiments. *, ** represent $p < 0.05$, $p < 0.01$, respectively, as obtained by the one-way ANOVA, followed by Fisher LSD multiple comparison tests.

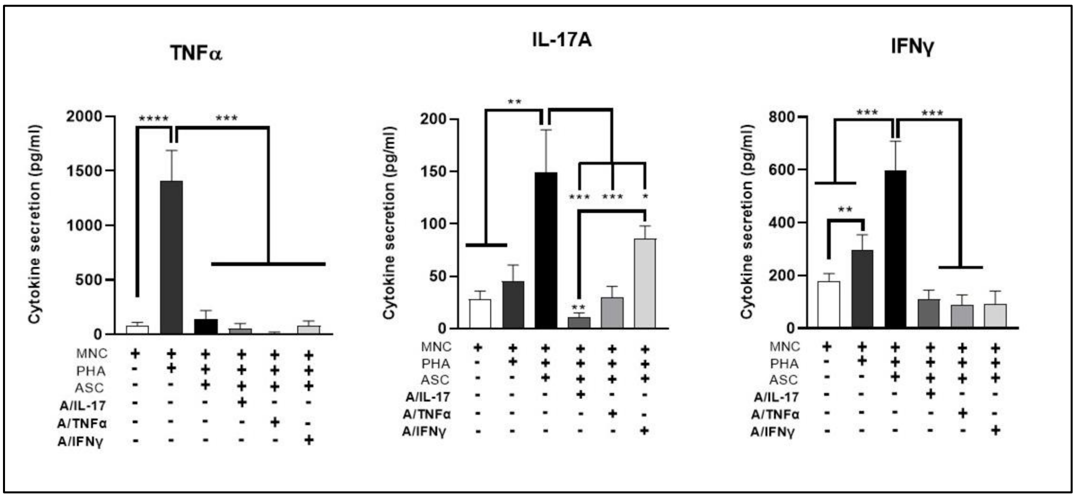

Supplementary Figure S5*. Neutralization of T cell cytokines in ob-ASC/MNC cocultures, and residual cytokine levels among CM*
MNC from healthy donors were activated or not with PHA (5µg/mL) and co-cultured with ob-ASC (5:1 ratio) (i) plus or minus A/IL-17A, (ii) A/ TNFα or (iii) A/ IFNγ) mAbs. 48h later, cytokine secretion was analyzed by ELISA for TNFα, IL-17A and IFNγ secretion. . *,**, *** ,****represent $p < 0.05$, $p < 0,01$, $p< 0,001$, $p<0.0001$, respectively, as obtained by the one way ANOVA followed by Fisher LSD multiple comparison tests.
